# Fatal cerebritis and ventriculitis secondary to tracheoesophageal prosthesis

**DOI:** 10.1093/jscr/rjae243

**Published:** 2024-04-18

**Authors:** Thomas J Crotty, Gerard P Sexton, Fergal Kavanagh, John Kinsella, Paul Lennon, Conrad V Timon, Conall W R Fitzgerald

**Affiliations:** Department of Otolaryngology–Head and Neck Surgery, St James’s Hospital, James Street, Dublin 8, Ireland; Department of Otolaryngology–Head and Neck Surgery, St James’s Hospital, James Street, Dublin 8, Ireland; Department of Otolaryngology–Head and Neck Surgery, St James’s Hospital, James Street, Dublin 8, Ireland; Department of Otolaryngology–Head and Neck Surgery, St James’s Hospital, James Street, Dublin 8, Ireland; Department of Otolaryngology–Head and Neck Surgery, St James’s Hospital, James Street, Dublin 8, Ireland; Department of Otolaryngology–Head and Neck Surgery, St James’s Hospital, James Street, Dublin 8, Ireland; Department of Otolaryngology–Head and Neck Surgery, St James’s Hospital, James Street, Dublin 8, Ireland

**Keywords:** larynx, head and neck surgery, oncology

## Abstract

Tracheoesophageal puncture and voice prosthesis placement is the preferred method of voice restoration following total laryngectomy. Although this is a safe and effective means of optimizing voice, severe complications can occur. We present the case of a patient who developed cerebritis and ventriculitis secondary to a tracheoesophageal prosthesis eroding his cervical vertebrae 20 years following pharyngo-laryngo-esophagectomy. Despite optimal antimicrobial therapy, he deteriorated and succumbed to his disease. Although tracheoesophageal prostheses are a safe and effective means of voice restoration, life-threatening complications can occur. This case report highlights a rare but severe case of cervical osteomyelitis, epidural abscess, and cerebritis and ventriculitis secondary to tracheoesophageal prosthesis. Clinicians must be aware of this severe complication in postlaryngectomy patients with tracheoesophageal prostheses.

## Introduction

Tracheoesophageal puncture and voice prosthesis placement allows laryngectomy patients to generate intelligible speech but requires continuous care and maintenance. Severe systemic infections secondary to tracheoesophageal prosthesis (TEP) are rare. We describe the case of cerebritis and pyogenic ventriculitis secondary to TEP.

## Case report

A 70-year-old male laryngectomy patient presented to the Emergency Department with a 3-day history of back pain, fever, and purulent discharge from his laryngectomy stoma. The patient was under routine clinical surveillance for 20 years following a pharyngo-laryngo-esophagectomy, gastric pull-up reconstruction, bilateral neck dissection, and adjuvant chemoradiotherapy for a pT4aN0M0 hypopharyngeal malignancy. Notably, the patient had recently been admitted to hospital with a spontaneous pharyngocutaneous fistula adjacent to his laryngectomy stoma, which was being managed conservatively with culture-directed antimicrobial therapy and wound care. Biopsy and magnetic resonance imaging (MRI) neck showed no evidence of a locoregional cancer recurrence.

On this presentation, the patient’s back pain localized to his cervical and upper thoracic spine. The patient denied experiencing a cough, shortness of breath, neck pain, or headache. There was no history of local trauma. Past medical history included type 2 diabetes mellitus, gout, hypothyroidism, and hypertension. The patient was not immunodeficient and demonstrated normal blood sugar levels and thyroid function tests. The patient had been undergoing regular Speech & Language Therapy review for TEP changes as required.

On examination, vital signs were normal and the patient was apyrexial. Clinical examination revealed purulent discharge from the TEP site and significant progression in the pharyngocutaneous fistula. There was no evidence of neck swelling or peristomal cellulitis. No evidence of focal neurology was noted with 5/5 power throughout his upper and lower limbs. Cardiorespiratory and abdominal exams were normal.

**Figure 1:**
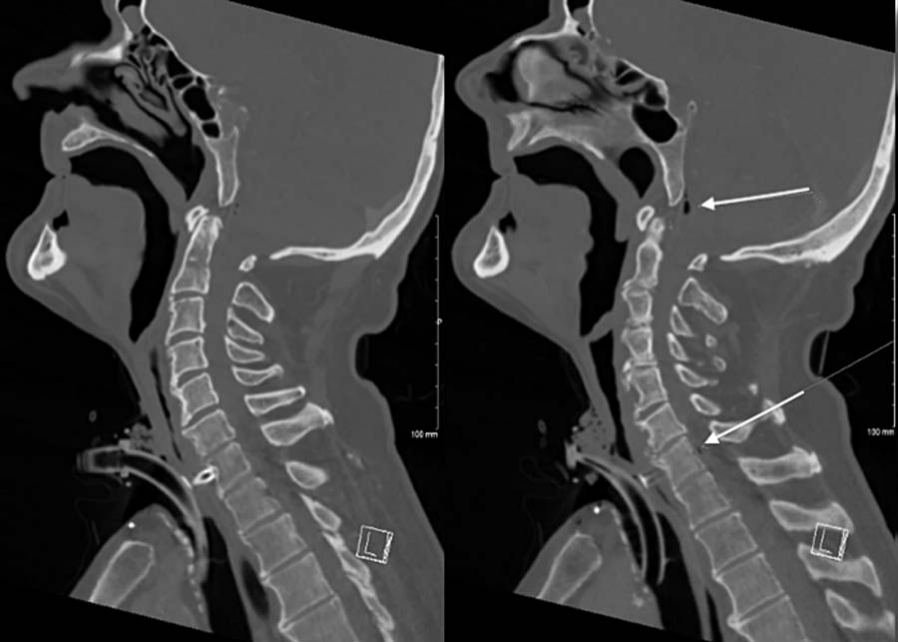
CT Neck demonstrating erosion of TEP into the C7 cervical resulting in osteomyelitis and a gas containing anterior epidural collection.

The patient’s C-reactive protein was 304 mg/dL (normal range, <5 mg/dL) and white cell count was 17 × 10^9^/L (normal range, 4.5–11 × 10^9^/L). Chest X-ray and urine dipstick detected no abnormalities. A contrast-enhanced computed tomography (CT) of the neck was performed demonstrating the erosion of the TEP through the posterior wall of the pharynx into the C7 vertebral body with osteomyelitis, an anterior epidural collection, and persistent pharyngocutaneous fistula (Fig. 1).

**Figure 2:**
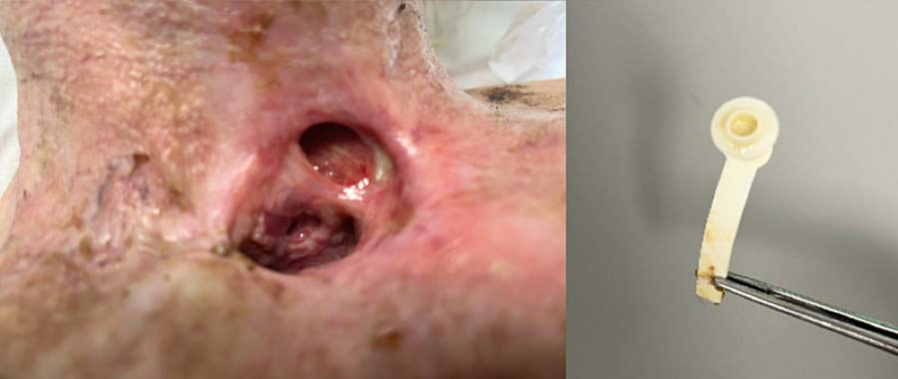
Clinical photograph of laryngectomy stoma and tracheoesophageal prosthesis.

**Figure 3:**
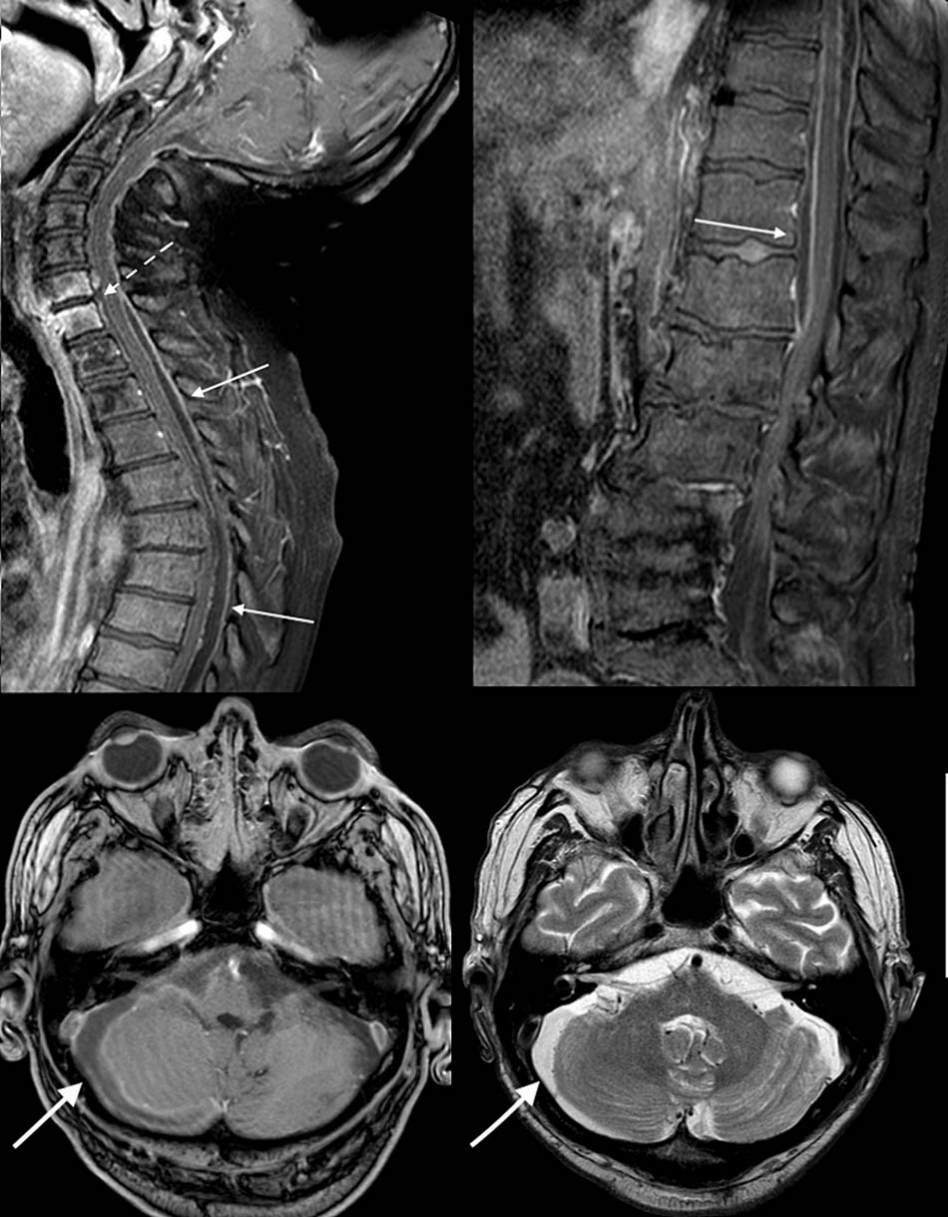
Initial MRI brain and whole spine demonstrating vertebral C6-C7 osteomyelitis (dotted arrow), subdural abscess within the cerebellum (bold arrow), and an epidural abscess within the spinal canal (arrow).

Urgent broad spectrum empiric antimicrobial therapy with Ceftriaxone, Metronidazole, and Vancomycin was initiated with guidance from Microbiology. Neurosurgical consultation advised MRI Brain and Whole Spine. In order to control the source of infection and obtain cultures and sensitivities, the TEP was removed under direct visualization (Fig. 2). A cuffed tracheostomy tube was inserted to prevent purulent discharge entering the trachea. MRI brain and whole spine revealed a vertebral C6-C7 osteomyelitis, subdural abscess, and epidural abscess within the spinal canal (Fig. 3). A recommendation for non-operative management with antimicrobial therapy was made by neurosurgery in light of the patient’s poor performance status and diffuse infection.

**Figure 4:**
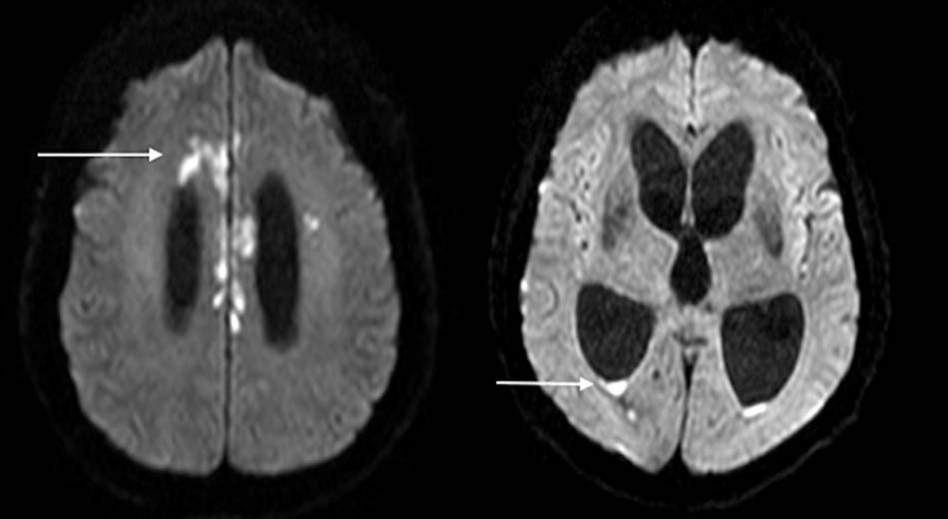
Diffusion-weighted MRI-brain showing intensely high signal in cerebral cortex (arrow) and ventricles (arrow) consistent with infective cerebritis and ventriculitis.

The patient became progressively obtunded, requiring intubation and ventilation in the Intensive Care Unit. Bacterial culture results demonstrated colonization with multiple microorganisms including *Staphylococcus aureus*, *Candida albicans*, *Proteus mirabilis*, *Klebsiella pneumoniae*, *Serratia marcescens*, and *Lactobacillus* species. This prompted the modification of antimicrobial coverage to Ceftazidime-Avabactam, Daptomycin, Metronidazole, and Vancomycin. Repeat imaging 10 days after presentation showed progression to diffuse cerebritis and pyogenic ventriculitis (Fig. 4). A multidisciplinary meeting was held with the patient’s next-of-kin and the decision was to provide comfort measures in light of the overall clinical picture, progression of infection, and existing poor patient performance status. The patient passed away peacefully in the presence of his family 13 days following his admission.

## Discussion

Tracheoesophageal puncture and TEP placement is associated with a low rate of severe complications [1]. Serious complications include aspiration pneumonia, TEP dislodgement, and deep neck space infections [2, 3]. While most infections are mild, TEPs can cause severe and life-threatening infections as seen in our case.

Two previous cases of epidural abscess secondary to TEP have been described [4, 5]. Ozturk *et al.* reported a case of a patient who developed a C6-C7 epidural abscess 9 years post-TEP placement. The patient, who underwent laryngectomy with adjuvant radiotherapy, presented with dysphagia, neck pain, and bilateral upper and lower extremity paresis. Despite surgical drainage and intravenous broad-spectrum antibiotics, the patient developed permanent quadriplegia. The second case report authored by Song *et al.* describes a patient with laryngeal adenoid cystic carcinoma treated with primary radiotherapy followed by salvage laryngectomy. They presented 4 months following TEP placement with progressive bilateral lower extremity weakness. MRI demonstrated an extensive epidural abscess from the level of C7 to T3 with associated osteomyelitis of the T1-T2 vertebral bodies. Considering the futility of further surgical intervention in the setting of progressive osteomyelitis and irreversible neurological deficits, a decision was made to initiate comfort measures.

The proposed mechanism for this complication is erosion of the prosthesis through the posterior pharyngeal/oesophageal wall leading to contamination of the retroesophageal space [6]. Further posterior displacement can cause erosion of the vertebrae leading to osteomyelitis and subsequent abscess development in the epidural space, as seen in Song *et al.* and our case. It is noteworthy that in all three previous cases, the patients had previously received radiation therapy. In two cases, the patients were many years posttreatment. Additionally, factors increasing the force directed on the posterior oesophageal wall by the prosthesis, such as excessive device length or digital pressure, are postulated to contribute to this complication [5–7]. Finally, TEP in the presence of a gastric pull-up reconstruction may increase the risk of migration or erosion due to the more flaccid, distensible nature of gastric tissue.

## Conclusion

Tracheoesophageal prosthesis is an acceptable form of speech rehabilitation in laryngectomy patients. Migration of TEP causing vertebral erosion and severe intracranial infection is a rare but highly morbid complication. Clinicians should have a high index of suspicion in laryngectomy patients presenting with non-specific neck or back pain, focal neurological deficits, or unexplained fever. Additionally, prior irradiation and inappropriate prosthesis length may increase the risk of developing this complication.
